# Factors associated with left ventricular reverse remodelling after percutaneous coronary intervention in patients with left ventricular systolic dysfunction

**DOI:** 10.1038/s41598-020-80491-y

**Published:** 2021-01-08

**Authors:** Yusuke Adachi, Arihiro Kiyosue, Jiro Ando, Takuya Kawahara, Satoshi Kodera, Shun Minatsuki, Hironobu Kikuchi, Toshiro Inaba, Hiroyuki Kiriyama, Kazutoshi Hirose, Hiroki Shinohara, Akihito Saito, Takayuki Fujiwara, Hironori Hara, Kazutaka Ueda, Kenichi Sakakura, Masaru Hatano, Mutsuo Harada, Eiki Takimoto, Hiroshi Akazawa, Hiroyuki Morita, Shin-ichi Momomura, Hideo Fujita, Issei Komuro

**Affiliations:** 1 Department of Cardiovascular Medicine, Graduate School of Medicine, The University of Tokyo, 7-3-1 Hongo, Bunkyo-ku, Tokyo, 113-8655 Japan; 2 Clinical Research Promotion Center, The University of Tokyo Hospital, Tokyo, Japan; 3 Division of Cardiovascular Medicine, Saitama Medical Center, Jichi Medical University, Saitama, Japan; 4 Department of Therapeutic Strategy for Heart Failure, Graduate School of Medicine, The University of Tokyo, Tokyo, Japan

**Keywords:** Cardiology, Interventional cardiology

## Abstract

Percutaneous coronary intervention (PCI) is sometimes considered as an alternative therapeutic strategy to surgical revascularization in patients with coronary artery disease (CAD) and reduced left ventricular ejection fraction (LVEF). However, the types or conditions of patients that receive the clinical benefit of left ventricular reverse remodelling (LVRR) remain unknown. The purpose of this study was to investigate the determinants of LVRR following PCI in CAD patients with reduced LVEF. From 4394 consecutive patients who underwent PCI, a total of 286 patients with reduced LV systolic function (LVEF < 50% at initial left ventriculography) were included in the analysis. LVRR was defined as LV end-systolic volume reduction ≥ 15% and improvement of LVEF ≥ 10% at 6 months follow-up left ventriculography. Patients were divided into LVRR (n = 63) and non-LVRR (n = 223) groups. Multivariate logistic regression analysis revealed that unprotected left main coronary artery (LMCA) intervention was significantly associated with LVRR (P = 0.007, odds ratios [OR] 4.70, 95% confidence interval [CI] 1.54–14.38), while prior PCI (P = 0.001, OR 0.35, 95% CI 0.19–0.66), presence of in-stent restenosis (P = 0.016, OR 0.32, 95% CI 0.12–0.81), and presence of de-novo stenosis (P = 0.038, OR 0.36, 95% CI 0.14–0.95) were negatively associated with LVRR. These data suggest the potential prognostic benefit of unprotected LMCA intervention for LVRR and importance of angiographic follow-up in patients with CAD and LV systolic dysfunction.

## Introduction

The increasing number of coronary artery disease (CAD) patients with reduced left ventricular (LV) systolic function is a major clinical problem^1^. Given LV systolic function is a powerful prognostic predictor in patients with CAD^1^, whether the revascularization therapy benefits patients by improving LV systolic function or not is an important clinical question to be answered when the therapy is considered.

While coronary artery bypass grafting (CABG) is thought to be a preferred therapy to treat CAD patients with reduced systolic function, percutaneous coronary intervention (PCI) has also become a strategy of choice in CAD patients with reduced systolic function^2,3^. Although PCI could improve LV function in some cases, it is not always effective and the types or conditions of patients that receive the benefits of LV functional improvement from revascularization by PCI remain unknown. LV functional improvement is typically associated with reduction in LV end-systolic volume, which is defined as LV reverse remodelling (LVRR). LVRR has been established as a prognostic factor in patients with ischemic or non-ischemic LV systolic dysfunction^4–6^. In the present study, we aim to examine the determinants of LVRR following PCI in CAD patients with reduced ejection fraction (EF).

## Methods

**Figure 1:**
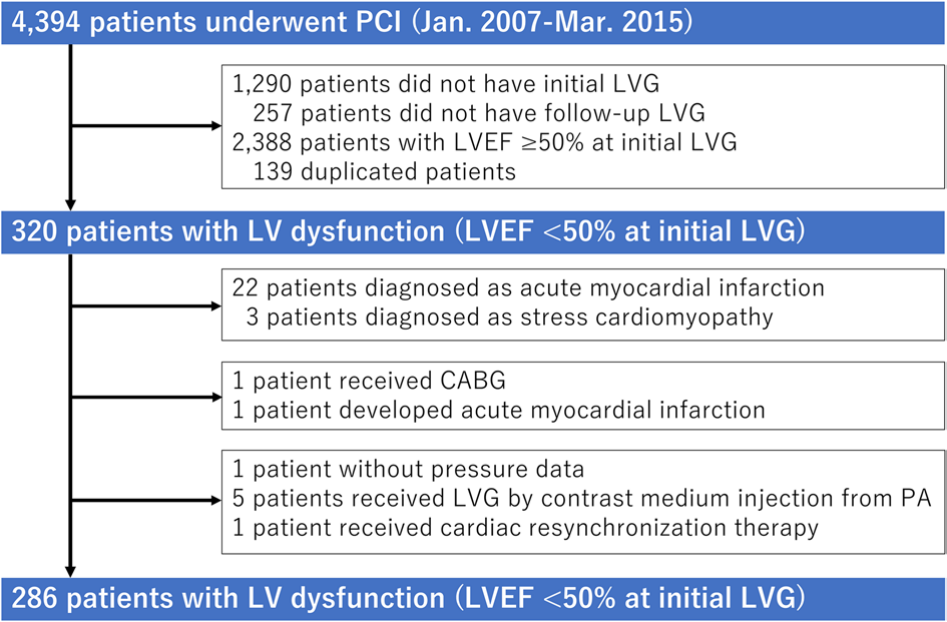
Flowchart of the study population. *PCI* percutaneous coronary intervention, *LVG* left ventriculography, *LVEF* left ventricular ejection fraction, *CABG* coronary artery bypass grafting, *PA* pulmonary artery.

We examined 4394 consecutive patients who underwent PCI in The University of Tokyo Hospital from January 2007 to March 2015. Left ventriculography (LVG) was routinely performed together with diagnostic coronary angiography (CAG) unless contraindicated (e.g., severe renal insufficiency, left ventricular thrombus, or elevated LV end-diastolic pressure). Follow-up CAG and LVG were also routinely performed approximately 6 months after each PCI. LVG performed together with the initial diagnostic CAG before PCI was defined as “initial LVG”, and LVG performed together with follow-up CAG after a series of PCI as “follow-up LVG”. At first, patients who did not have initial LVG (n = 1290) were excluded. Then, patients who did not have follow-up LVG (n = 257) were excluded. Next, patients with an LVEF ≥ 50% at initial LVG (n = 2388) were also excluded. Duplicated counting of patient number due to multiple procedures during one LVG follow-up period was corrected (n = 139). Furthermore, we excluded patients diagnosed as acute myocardial infarction (n = 22) and stress cardiomyopathy (n = 3). Acute myocardial infarction was defined as a rise in cardiac enzyme concentrations (troponin I and/or T, and/or creatine phosphokinase-MB) with at least one value above the 99th percentile upper reference limit in the first 24 h and with at least one of the following: (1) symptoms of myocardial ischemia, (2) new or presumed new significant ST-segment-T wave changes or left bundle branch block, (3) development of pathological Q waves in electrocardiogram, (4) imaging evidence of new loss of viable myocardium or a new regional wall motion abnormality, and (5) identification of an intracoronary thrombus by angiography^7^. Subacute myocardial infarction (≤ 1 week from onset) was also considered as acute myocardial infarction, whereas recent myocardial infarction (> 1 week from onset or unknown onset) were considered as old myocardial infarction, and were included in the present study. In addition, one patient who underwent CABG (n = 1) after PCI and one patient who newly developed acute myocardial infarction (n = 1) during the follow-up period were also excluded. A patient without pressure data during the LVG study (n = 1) and patients who received LVG by contrast medium injection from the pulmonary artery because of left ventricular thrombus (n = 5) were excluded. A patient who received cardiac resynchronization therapy during the follow-up period was excluded from the analysis, which may itself affect LVRR (n = 1). There were no patients included who newly received implantable cardioverter defibrillators or cardiac resynchronization therapy during the study. Finally, a total of 286 patients with CAD and LV dysfunction (LVEF < 50% at initial LVG) who underwent PCI and received follow-up LVG were included in the present study (Fig. 1).

All LVG data analysis was performed by skilled radiology technicians in our catheterization laboratory at the time of LVG, and was approved by experienced cardiologists. LVG was performed during retrograde left heart catheterization with the patient in the 30° right anterior oblique position^8^. End-diastolic and end-systolic endocardial contours were traced in the frames with maximal and minimum volume, respectively. Left ventricular volume was calculated by the area-length method. LVEF% was calculated from end-diastolic and end-systolic left ventricular volumes (LVEDV and LVESV, respectively) as (LVEDV − LVESV) × 100/LVEDV^9^. Because the aim of our study was to investigate the determinants of LVRR, it was important to compare LVG findings before and after PCI. LVRR was defined as LV end-systolic volume reduction ≥ 15% and improvement of LVEF ≥ 10%^10^.

We typically followed the current guidelines when patients were referred for revascularization^2,3^. Revascularization by PCI was considered for stable angina patients who have angiographically significant stenosis (≥ 75% diameter stenosis of a major epicardial artery segment^11^, or ≥ 50% diameter stenosis in the left main coronary artery^12^) or functionally significant stenosis (fractional flow reserve measurement < 0.80). Coronary in-stent restenosis was defined as the angiographic detection of a recurrent stenosis with a diameter > 50% at the stent segment or its 5 mm adjacent segments^13^. Unprotected left main coronary artery (LMCA) disease was defined as angiographically significant stenosis of the LMCA without patent surgical grafts to the left coronary artery system^14^.

Hypertension was defined as systolic blood pressure > 140 mmHg, diastolic blood pressure > 90 mmHg, or medical treatment for hypertension on admission. Diabetes mellitus was defined as a haemoglobin A1c level in National Glycohemoglobin Standardization Program (NGSP) units > 6.5% or medical treatment for diabetes mellitus. If the haemoglobin A1c level in Japan Diabetes Society (JDS) units was only available, a conversion from HbA1c (JDS) to HbA1c (NGSP) units was performed as follows: NGSP (%) = 1.02 × JDS (%) + 0.25%^15^. Dyslipidaemia was defined as a low-density lipoprotein cholesterol level > 140 mg/dl or treatment for dyslipidaemia. Chronic kidney disease was defined as glomerular filtration rate < 60 mL/min/1.73 m^2^. Hyperuricemia was defined as serum uric acid > 7.0 mg/dL or treatment for hyperuricemia. The LMCA was defined as segment from the ostium of the left coronary artery through the bifurcation into the left anterior descending and left circumflex branches. The proximal left anterior descending artery (LAD) was defined as a segment of the LAD proximal to and including the first major septal branch. The proximal left circumflex artery (LCx) was defined as the main stem of the circumflex from its origin of the left main and including the origin of the first obtuse marginal branch. The proximal right coronary artery (RCA) was defined as the segment from the ostium to half the distance to the acute margin of the heart^16^. A chronic total occlusion was defined as a complete coronary arterial obstruction with thrombolysis in myocardial infarction flow grade of 0 for > 3 months (estimated)^17^. The present study was conducted according to the guidelines of the Declaration of Helsinki and was approved by the institutional research ethics committee of the University of Tokyo (Approval Number 2650). The informed consent was waived due to the retrospective nature of the study according to the ethics committee approval.

### Statistical analysis

All study patients were divided into LVRR and non-LVRR groups. We compared patient characteristics, LVG findings, types of intervention, and medications at discharge after PCI between the groups. Categorical variables are presented as numbers (percentages), and were compared using the chi-squared test. Continuous variables are expressed as the mean ± standard deviation or median [interquartile range], depending on their distribution as assessed by visual inspection and the Shapiro–Wilk test. Normally distributed continuous variables were compared using the Student’s *t* test. Otherwise, continuous variables were compared using the Mann–Whitney U-test. Univariate and multivariate logistic regression analyses were applied to investigate the determinants of LVRR. In these logistic regression models, the dependent variable was LVRR. Variables that showed a marginal association with LVRR (P < 0.10) in the univariate logistic regression analyses, use of angiotensin converting enzyme inhibitor (ACE-I)^18,19^ and/or angiotensin II receptor blocker (ARB)^20^, and use of beta-blocker^21–23^ were adopted as independent variables. Odds ratios (OR) and 95% confidence intervals (CI) were calculated. A value of P < 0.05 was considered statistically significant. All analyses were performed using statistical software (SPSS v20; SPSS, Inc., Chicago, IL, USA).

## Results

A total of 286 patients were divided into the LVRR (n = 63) and the non-LVRR (n = 223) groups. The baseline characteristics of the study patients are shown in Table 1. The mean age of the LVRR group (66.8 ± 7.8 years) was similar to that of the non-LVRR group (67.0 ± 9.1; P = 0.90). The prevalence of male sex in the LVRR group (76.2%) was significantly lower than that in the non-LVRR group (87.0%; P = 0.047). The prevalence of prior PCI in the LVRR group (36.5%) was significantly lower than that in the non-LVRR group (63.2%; P < 0.001).

**Table 1:** Patient characteristics.

	All (n = 286)	LVRR group (n = 63)	Non-LVRR group (n = 223)	P value
Age (years)	66.9 ± 8.8	66.8 ± 7.8	67.0 ± 9.1	0.90
Male sex, n (%)	242 (84.6)	48 (76.2)	194 (87.0)	0.047
BMI (kg/m^2^)	24.0 ± 3.3	23.9 ± 3.4	24.0 ± 3.3	0.68
Hypertension, n (%)	267 (93.4)	58 (92.1)	209 (93.7)	0.58
Diabetes mellitus, n (%)	156 (54.5)	29 (46.0)	127 (57.0)	0.15
Dyslipidaemia, n (%)	235 (82.2)	50 (79.4)	185 (83.0)	0.57
Chronic kidney disease, n (%)	99 (34.6)	22 (34.9)	77 (34.5)	1.00
Stage 3A, n (%)	51 (17.8)	11 (17.5)	40 (17.9)	
Stage 3B, n (%)	3 (1.0)	0 (0)	3 (1.3)	
Stage 4, n (%)	0 (0)	0 (0)	0 (0)	
Stage 5 (on haemodialysis), n (%)	45 (15.7)	11 (17.5)	34 (15.2)	
Hyperuricemia, n (%)	70 (24.5)	18 (28.6)	52 (23.3)	0.41
Stroke, n (%)	39 (13.6)	10 (15.9)	29 (13.0)	0.68
Chronic obstructive pulmonary disease, n (%)	7 (2.4)	1 (1.6)	6 (2.7)	0.70
Smoking, n (%)	207 (72.4)	44 (69.8)	163 (73.1)	0.63
Atrial fibrillation, n (%)	22 (7.7)	3 (4.8)	19 (8.5)	0.43
Moderate aortic valve stenosis, n (%)	4 (1.4)	2 (3.2)	2 (0.9)	0.21
Severe aortic valve stenosis, n (%)	0 (0)	0 (0)	0 (0)	-
Moderate to severe aortic valve regurgitation, n (%)	0 (0)	0 (0)	0 (0)	-
Family history of coronary artery disease, n (%)	74 (25.9)	13 (20.6)	61 (27.4)	0.33
Previous acute coronary syndrome, n (%)	200 (69.9)	41 (65.1)	159 (71.3)	0.28
STEMI, n (%)	189 (66.1)	41 (65.1)	148 (66.4)	
NSTEMI (without history of STEMI), n (%)	4 (1.4)	0 (0)	4 (1.8)	
UA (without history of STEMI or NTEMI), n (%)	7 (2.4)	0 (0)	7 (3.1)	
Prior PCI, n (%)	164 (57.3)	23 (36.5)	141 (63.2)	< 0.001
Prior CABG, n (%)	32 (11.2)	4 (6.3)	28 (12.6)	0.19
Prior permanent pacemaker implantation, n (%)	3 (1.0)	0 (0)	3 (1.3)	0.60
Prior implantable cardioverter defibrillator, n (%)	3 (1.0)	0 (0)	3 (1.3)	0.60
Prior cardiac resynchronization therapy, n (%)	1 (0.3)	0 (0)	1 (0.4)	1.00

Data are expressed as mean ± standard deviation or numbers (%).

*LVRR* left ventricular reverse remodelling; *BMI* body mass index; *STEMI* ST-elevation myocardial infarction; *NSTEMI* non-ST-elevation myocardial infarction; *UA* unstable angina; *PCI* percutaneous coronary intervention; *CABG* coronary artery bypass grafting.

LVG findings of the study patients are shown in Table 2. Median [interquartile range] follow up (i.e. intervals between initial LVG and follow-up LVG) were 7 [6–8] months in both groups. In the initial LVG, LVEF, LVEDV, LVESV, LVED volume index (LVEDVI), and LVESVI were similar between the LVRR and non-LVRR groups. LV systolic pressure was significantly higher in the LVRR group (129.1 ± 24.8 mmHg) compared with the non-LVRR group (119.4 ± 24.4 mmHg; P = 0.006). LVED pressure was similar between the LVRR and non-LVRR groups. Aortic systolic pressure was significantly higher in the LVRR group (134.7 ± 24.5 mmHg) compared with the non-LVRR group (122.7 ± 24.7 mmHg; P = 0.001). Aortic diastolic pressure was also significantly higher in the LVRR group (65.5 ± 12.4 mmHg) compared with the non-LVRR group (61.3 ± 11.8 mmHg; P = 0.015). Heart rate, mitral regurgitation grade, Canadian Cardiovascular Society grading of angina pectoris, haemoglobin level, and B-type natriuretic peptide level were similar between the LVRR and non-LVRR groups at initial LVG.

**Table 2:** Left ventriculography (LVG) findings.

	All (n = 286)	LVRR group (n = 63)	Non-LVRR group (n = 223)	P value
**Initial LVG**				
LVEF (%)	44.4 [39.3–47.9]	43.7 [37.1–47.6]	44.8 [39.8–48.0]	0.20
LVEDV (mL)	119.7 [91.1–152.0]	119.6 [98.4–149.8]	119.8 [90.9–152.7]	0.93
LVESV (mL)	66.8 [51.0–85.7]	66.6 [52.4–87.0]	66.9 [50.7–85.2]	0.71
LVEDVI (mL/m^2^)	72.1 [54.7–87.4]	72.9 [55.6–90.4]	71.6 [53.9–87.1]	0.49
LVESVI (mL/m^2^)	39.3 [30.0–50.7]	41.6 [30.3–52.7]	38.9 [30.0–49.2]	0.35
LV systolic pressure (mmHg)	121.5 ± 24.8	129.1 ± 24.8	119.4 ± 24.4	0.006
LVED pressure (mmHg)	9 [6–13]	8 [6–12]	9 [6–13]	0.29
Aortic systolic pressure (mmHg)	125.3 ± 25.1	134.7 ± 24.5	122.7 ± 24.7	0.001
Aortic diastolic pressure (mmHg)	62.3 ± 12.1	65.5 ± 12.4	61.3 ± 11.8	0.015
Heart rate at LVG (beats/min)	66 [60–75]	68 [60–77]	65 [60–74]	0.18
**Mitral regurgitation grade**				0.75
Sellers I or no regurgitation, n (%)	229 (80.1)	49 (77.8)	180 (80.7)	
Sellers II, n (%)	51 (17.8)	12 (19.0)	39 (17.5)	
Sellers III, n (%)	6 (2.1)	2 (3.2)	4 (1.8)	
Sellers IV, n (%)	0 (0)	0 (0)	0 (0)	
**Canadian Cardiovascular Society grading of angina pectoris**				0.54
I, n (%)	180 (62.9)	35 (55.6)	145 (65.0)	
II, n (%)	86 (30.1)	22 (34.9)	64 (28.7)	
III, n (%)	17 (5.9)	5 (7.9)	12 (5.4)	
IV, n (%)	3 (1.0)	1 (1.6)	2 (0.9)	
Haemoglobin (g/dL)	13.1 ± 1.7	12.8 ± 1.9	13.1 ± 1.7	0.14
B-type natriuretic peptide (pg/mL)	64.5 [29.6–183.2]	61.8 [19.3–237.2]	65.6 [30.0–165.8]	0.74
**Follow-up LVG**				
LVEF (%)	49.0 ± 9.7	59.5 ± 6.4	46.0 ± 8.3	< 0.001
LVEDV (mL)	116.0 [89.8–147.3]	93.0 [72.2–127.7]	121.3 [96.5–151.0]	< 0.001
LVESV (mL)	58.0 [43.0–78.8]	35.7 [27.8–53.1]	64.4 [48.2–85.8]	< 0.001
LVEDVI (mL/m^2^)	67.8 [53.1–87.6]	56.3 [44.9–74.7]	72.2 [56.4–90.4]	< 0.001
LVESVI (mL/m^2^)	33.7 [25.4–46.0]	21.9 [17.1–30.7]	37.7 [29.4–48.6]	< 0.001
LV systolic pressure (mmHg)	118.7 ± 23.3	123.6 ± 22.0	117.2 ± 23.5	0.054
LVED pressure (mmHg)	8 [6–12]	8 [6–11]	9 [6–12]	0.14
Aortic systolic pressure (mmHg)	120.4 ± 23.5	125.8 ± 22.7	118.8 ± 23.6	0.037
Aortic diastolic pressure (mmHg)	59.4 ± 12.0	60.2 ± 11.5	59.2 ± 12.2	0.55
Heart rate at LVG (beats/min)	66 [58–73.25]	68 [60–76]	66 [57–73]	0.26
**Mitral regurgitation grade**				0.91
Sellers I or no regurgitation, n (%)	235 (82.2)	53 (84.1)	182 (81.6)	
Sellers II, n (%)	49 (17.1)	10 (15.9)	39 (17.5)	
Sellers III, n (%)	2 (0.7)	0 (0)	2 (0.9)	
Sellers IV, n (%)	0 (0)	0 (0)	0 (0)	
**Canadian Cardiovascular Society grading of angina pectoris**				0.87
I, n (%)	254 (88.8)	57 (90.5)	197 (88.3)	
II, n (%)	22 (7.7)	5 (7.9)	17 (7.6)	
III, n (%)	8 (2.8)	1 (1.6)	7 (3.1)	
IV, n (%)	2 (0.7)	0 (0)	2 (0.9)	
Haemoglobin (g/dL)	12.9 ± 1.6	12.7 ± 1.7	13.0 ± 1.6	0.18
B-type natriuretic peptide (pg/mL)	61.5 [26.8–144.4]	41.6 [23.5–112.8]	69.5 [27.8–148.2]	0.042
Intervals between initial LVG and follow-up LVG (month)	7 [6–8]	7 [6–8]	7 [6–8]	0.38
Intervals between last PCI and follow-up LVG (month)	6 [6–7]	6 [6–7]	6 [6–7]	0.23

Data are expressed as numbers (%), mean ± standard deviation, or median [interquartile range].

*LVEF* left ventricular ejection fraction; *LVEDV* left ventricular end-diastolic volume; *LVESV* left ventricular end-systolic volume; *LVEDVI* left ventricular end-diastolic volume index; *LVESVI* left ventricular end-systolic volume index.

At follow-up LVG, LVEF was significantly higher in the LVRR group (59.5 ± 6.4%) compared with the non-LVRR group (46.0 ± 8.3%; P < 0.001). LVEDV, LVESV, LVEDVI, and LVESVI were significantly lower in the LVRR group (all P < 0.001). B-type natriuretic peptide level at follow-up LVG was significantly lower in the LVRR group (41.6 [23.5–112.8] pg/mL) compared with the non-LVRR group (69.5 [27.8–148.2] pg/mL; P = 0.042), which may reflect the reverse remodelling of left ventricle. LV systolic pressure showed a trend towards being higher in the LVRR group (123.6 ± 22.0 mmHg) compared with the non-LVRR group (117.2 ± 23.5 mmHg; P = 0.054). Aortic systolic pressure was significantly higher in the LVRR group (125.8 ± 22.7 mmHg) compared with the non-LVRR group (118.8 ± 23.6 mmHg; P = 0.037). LVED pressure, aortic diastolic pressure, heart rate, mitral regurgitation grade, Canadian Cardiovascular Society grading of angina pectoris, and haemoglobin level were similar between the LVRR group and the non-LVRR group at follow-up LVG.

Details of the PCIs are shown in Table 3. Target vessels were divided into seven groups: unprotected LMCA, LAD (proximal) without LMCA intervention, LAD (middle to distal) without proximal intervention, LCx (proximal) without LMCA intervention, LCx (middle to distal) without proximal intervention, RCA (proximal), and RCA (middle to distal and posterior interventricular branch) without proximal intervention. The prevalence of unprotected LMCA intervention in the LVRR group (14.3%) was significantly higher than that in the non-LVRR group (4.0%; P = 0.006). The prevalence of PCIs in other vessels were similar between the LVRR and non-LVRR groups. The frequency of use of a sirolimus-eluting stent, everolimus-eluting stent, paclitaxel-eluting stent, Endeavor zotarolimus-eluting stent, Resolute zotarolimus-eluting stent, biolimus-eluting stent with biodegradable polymer, biodegradable-polymer sirolimus-eluting stent, bare-metal stent, drug-coated balloon, plain old balloon angioplasty without stenting, atherectomy, and intracoronary imaging were also similar between the LVRR and non-LVRR groups. The prevalence of PCI to chronic total occlusion and presence of residual stenosis were also similar between the LVRR and non-LVRR groups. The prevalence of in-stent restenosis at follow-up angiography in the LVRR group (11.1%) was significantly less than that in the non-LVRR group (24.2%; P = 0.035). The prevalence of de novo stenosis at follow-up angiography in the LVRR group (9.5%) was significantly less than that in the non-LVRR group (23.8%; P = 0.021).

**Table 3:** Percutaneous coronary intervention.

	All (n = 286)	LVRR group (n = 63)	Non-LVRR group (n = 223)	P value
**Target vessel**				
Unprotected LMCA, n (%)	18 (6.3)	9 (14.3)	9 (4.0)	0.006
LAD (proximal) without LMCA intervention, n (%)	109 (38.1)	26 (41.3)	83 (37.2)	0.66
LAD (middle to distal) without proximal intervention, n (%)	46 (16.1)	9 (14.3)	37 (16.6)	0.70
LCx (proximal) without LMCA intervention, n (%)	34 (11.9)	7 (11.1)	27 (12.1)	1.000
LCx (middle to distal) without proximal intervention, n (%)	53 (18.5)	12 (19.0)	41 (18.4)	1.000
RCA (proximal), n (%)	93 (32.5)	20 (31.7)	73 (32.7)	1.000
RCA (middle to distal and posterior interventricular branch) without proximal intervention, n (%)	128 (44.8)	26 (41.3)	102 (45.7)	0.57
**Type of stent (or drug-coated balloon) used**				
Sirolimus-eluting stent, n (%)	81 (28.3)	22 (34.9)	59 (26.5)	0.21
Total number of stents	151	42	109	
Everolimus-eluting stent, n (%)	124 (43.4)	28 (44.4)	96 (43.0)	0.89
Total number of stents	279	70	209	
Paclitaxel-eluting stent, n (%)	22 (7.7)	6 (9.5)	16 (7.2)	0.59
Total number of stents	38	10	28	
Endeavor zotarolimus-eluting stent, n (%)	4 (1.4)	0 (0)	4 (1.8)	0.58
Total number of stents	4	0	4	
Resolute zotarolimus-eluting stent, n (%)	12 (4.2)	4 (6.3)	8 (3.6)	0.47
Total number of stents	15	6	9	
Biolimus-eluting stent with biodegradable polymer, n (%)	31 (10.8)	6 (9.5)	25 (11.2)	0.82
Total number of stents	46	8	38	
Biodegradable-polymer sirolimus-eluting stent, n (%)	1 (0.4)	1 (1.6)	0 (0)	0.22
Total number of stents	2	2	0	
Bare-metal stent, n (%)	24 (8.4)	3 (4.8)	21 (9.4)	0.31
Total number of stents	33	3	30	
Drug-coated balloon, n (%)	6 (2.1)	0 (0)	6 (2.7)	0.35
Total number of sites	8	0	8	
Plain old balloon angioplasty without stenting, n (%)	54 (18.9)	8 (12.7)	46 (20.6)	0.20
Total number of sites	57	8	49	
Use of atherectomy, n (%)	18 (6.3)	3 (4.8)	15 (6.7)	0.77
Use of intracoronary imaging, n (%)	277 (96.9)	63 (100.0)	214 (96.0)	0.21
Intravascular ultrasound	275 (96.2)	63 (100.0)	212 (95.1)	
Optical coherence tomography	4 (1.4)	0 (0)	4 (1.8)	
Intravascular ultrasound + optical coherence tomography	2 (0.7)	0 (0)	2 (0.9)	
PCI to chronic total occlusion, n (%)	37 (12.9)	6 (9.5)	31 (13.9)	0.41
Presence of residual stenosis, n (%)	187 (65.4)	38 (60.3)	149 (66.8)	0.37
Presence of in-stent restenosis at follow-up angiography, n (%)	61 (21.3)	7 (11.1)	54 (24.2)	0.035
Presence of de novo stenosis at follow-up angiography, n (%)	59 (20.6)	6 (9.5)	53 (23.8)	0.021

Data are expressed as numbers (%).

*LMCA* left main coronary artery; *LAD* left anterior descending artery; *LCx* left circumflex artery; *RCA* right coronary artery.

Details of medications at discharge after PCI are shown in Table 4. The prescription rate of ACE-I and/or ARB was similar between the LVRR (84.1%) and non-LVRR (84.3%; P = 1.00) groups. The prescription rate of beta-blocker was also similar between the LVRR (71.4%) and non-LVRR (70.4%; P = 0.88) groups. Furthermore, the prescription rate of other drugs was similar between the LVRR and non-LVRR groups. Angiotensin receptor neprilysin inhibitors were not available during the study period which are now considered as Class I drugs in systolic heart failure. Details of medications on admission of initial LVG are shown in supplemental Table S1.

**Table 4:** Medications at discharge after PCI.

	All (n = 286)	LVRR group (n = 63)	Non-LVRR group(n = 223)	P value
ACE-I and/or ARB, n (%)	241 (84.3)	53 (84.1)	188 (84.3)	1.00
Beta-blocker, n (%)	202 (70.6)	45 (71.4)	157 (70.4)	0.88
Direct renin inhibitor, n (%)	2 (0.7)	1 (1.6)	1 (0.4)	0.39
Mineralocorticoid receptor antagonist, n (%)	24 (8.4)	4 (6.3)	20 (9.0)	0.62
Spironolactone, n (%)	23 (8.0)	4 (6.3)	19 (8.5)	0.79
Eplerenone, n (%)	1 (0.3)	0 (0)	1 (0.4)	1.00
Class I antiarrhythmic agent, n (%)	12 (4.2)	1 (1.6)	11 (4.9)	0.31
Class III antiarrhythmic agent, n (%)	5 (1.7)	0 (0)	5 (2.2)	0.36
Calcium channel blocker, n (%)	108 (37.8)	29 (46.0)	79 (35.4)	0.14
Dihydropyridine, n (%)	95 (33.2)	26 (41.3)	69 (30.9)	0.13
Non-dihydropyridine, n (%)	16 (5.6)	3 (4.8)	13 (5.8)	0.78
Nitric acid, n (%)	63 (22.0)	16 (25.4)	47 (21.1)	0.49
Nicorandil, n (%)	115 (40.2)	25 (39.7)	90 (40.4)	1.00
Alpha-blocker, n (%)	4 (1.4)	2 (3.2)	2 (0.9)	0.21
Diuretic agent, n (%)	59 (20.6)	13 (20.6)	46 (20.6)	1.00
Digitalis, n (%)	11 (3.8)	3 (4.8)	8 (3.6)	0.71
Orally active cardiac stimulant, n (%)	0 (0)	0 (0)	0 (0)	-
Anticoagulant agent, n (%)	39 (13.6)	6 (9.5)	33 (14.8)	0.31
Warfarin, n (%)	38 (13.3)	5 (7.9)	33 (14.8)	0.21
Direct oral anticoagulant, n (%)	1 (0.3)	1 (1.6)	0 (0)	0.22
Statin, n (%)	244 (85.3)	55 (87.3)	189 (84.8)	0.69
Oral hypoglycaemic agent, n (%)	109 (38.1)	19 (30.2)	90 (40.4)	0.15
Insulin, n (%)	54 (18.9)	15 (23.8)	39 (17.5)	0.28

Data are expressed as numbers (%).

*ACE-I* angiotensin converting enzyme inhibitor; *ARB* angiotensin II receptor blocker.

The results of the univariate and multivariate logistic regression analyses are shown in Table 5. Unprotected LMCA intervention (P = 0.007, OR 4.70, 95% CI 1.54–14.38) was significantly associated with LVRR, while prior PCI (P = 0.001, OR 0.35, 95% CI 0.19–0.66), presence of in-stent restenosis (P = 0.016, OR 0.32, 95% CI 0.12–0.81), and presence of de novo stenosis (P = 0.038, OR 0.36, 95% CI 0.14–0.95) were negatively associated with LVRR in this multivariate analysis.

**Table 5:** Determinants of LVRR: univariate and multivariate logistic regression analysis.

Variables	Univariate logistic regression analysis			Multivariate logistic regression analysis		
	OR	95% CI	P value	OR	95% CI	P value
Age, every 10 years	0.98	0.71–1.35	0.90			
Male	0.48	0.24–0.96	0.039	0.70	0.31–1.58	0.39
BMI, every 1 kg/m^2^	0.98	0.90–1.07	0.68			
Hypertension	0.78	0.27–2.25	0.64			
Diabetes mellitus	0.65	0.37–1.13	0.13			
Dyslipidaemia	0.77	0.38–1.56	0.47			
Chronic kidney disease (stage 3–5)	1.02	0.57–1.83	0.95			
Hyperuricemia	0.94	0.41–2.16	0.88			
Stroke	1.26	0.58–2.75	0.56			
Chronic obstructive pulmonary disease	0.58	0.069–4.94	0.62			
Smoking	0.84	0.45–1.55	0.57			
Atrial fibrillation	0.53	0.15–1.87	0.33			
Moderate aortic valve stenosis	3.62	0.50–26.25	0.20			
Family history of coronary artery disease	0.69	0.35–1.36	0.28			
Previous acute coronary syndrome	0.75	0.41–1.36	0.34			
Prior PCI	0.33	0.19–0.60	< 0.001	0.35	0.19–0.66	0.001
Prior CABG	0.47	0.16–1.40	0.18			
Initial LVG						
LV systolic pressure, every 10 mmHg	1.17	1.04–1.31	0.007	1.03	0.77–1.36	0.86
LVED pressure, every 1 mmHg	0.96	0.91–1.03	0.25			
Aortic systolic pressure, every 10 mmHg	1.21	1.08–1.36	0.001	1.18	0.89–1.58	0.25
Aortic diastolic pressure, every 10 mmHg	1.33	1.05–1.68	0.016	1.09	0.80–1.47	0.60
Heart rate at LVG, every 10 beats/min	1.16	0.96–1.40	0.14			
Mitral regurgitation grade						
Sellers II (vs. Sellers I or no regurgitation)	1.13	0.55–2.32	0.74			
Sellers III (vs. Sellers I or no regurgitation)	1.84	0.33–10.32	0.49			
Canadian Cardiovascular Society grading of angina pectoris						
II (vs. I)	1.42	0.78–2.62	0.26			
III (vs. I)	1.73	0.57–5.22	0.33			
IV (vs. I)	2.07	0.18–23.50	0.56			
Haemoglobin, every 1 g/dL	0.89	0.76–1.04	0.15			
B-type natriuretic peptide, every 100 pg/mL	1.01	0.96–1.06	0.79			
Follow-up LVG						
LV systolic pressure, every 10 mmHg	1.12	1.00–1.26	0.056	0.98	0.73–1.32	0.91
LVED pressure, every 1 mmHg	0.95	0.89–1.02	0.15			
Aortic systolic pressure, every 10 mmHg	1.13	1.01–1.27	0.039	0.96	0.71–1.29	0.76
Aortic diastolic pressure, every 10 mmHg	1.07	0.85–1.35	0.55			
Heart rate at LVG, every 10 beats/min	1.11	0.88–1.40	0.38			
Mitral regurgitation grade						
Sellers II (vs. Sellers I or no regurgitation)	0.88	0.41–1.88	0.74			
Canadian Cardiovascular Society grading of angina pectoris						
II (vs. I)	1.02	0.36–2.88	0.98			
III (vs. I)	0.49	0.060–4.10	0.51			
Haemoglobin, every 1 g/dL	0.89	0.74–1.06	0.19			
B-type natriuretic peptide, every 100 pg/mL	0.94	0.85–1.04	0.24			
Intervals between initial LVG and follow-up LVG, every 1 month	1.00	0.94–1.06	0.96			
Intervals between last PCI and follow-up LVG, every 1 month	1.00	0.94–1.06	0.98			
**Target vessel**						
Unprotected LMCA	3.96	1.50–10.46	0.005	4.70	1.54–14.38	0.007
LAD (proximal) without LMCA intervention	1.19	0.67–2.10	0.56			
LAD (middle to distal) without proximal intervention	0.84	0.38–1.84	0.66			
LCx (proximal) without LMCA intervention	0.91	0.38–2.19	0.83			
LCx (middle to distal) without proximal intervention	1.04	0.51–2.13	0.91			
RCA (proximal)	0.96	0.53–1.74	0.88			
RCA (middle to distal and posterior interventricular branch) without proximal intervention	0.83	0.47–1.47	0.53			
**Type of stent used**						
Sirolimus-eluting stent, every 1 stent	1.16	0.91–1.49	0.23			
Everolimus-eluting stent, every 1 stent	1.07	0.90–1.28	0.43			
Paclitaxel-eluting stent, every 1 stent	1.13	0.68–1.88	0.65			
Resolute zotarolimus-eluting stent, every 1 stent	1.89	0.77–4.64	0.17			
Biolimus-eluting stent with biodegradable polymer, every 1 stent	0.84	0.47–1.53	0.57			
Bare-metal stent, every 1 stent	0.48	0.17–1.39	0.18			
Plain old balloon angioplasty without stenting, every 1 site	0.55	0.25–1.20	0.13			
Use of atherectomy	0.69	0.19–2.48	0.57			
PCI to chronic total occlusion	0.65	0.26–1.64	0.36			
Presence of residual stenosis	0.76	0.42–1.34	0.34			
Presence of in-stent restenosis at follow-up angiography	0.39	0.17–0.91	0.029	0.32	0.12–0.81	0.016
Presence of de novo stenosis at follow-up angiography	0.34	0.14–0.83	0.018	0.36	0.14–0.95	0.038
Medication						
ACE-I and/or ARB	0.99	0.46–2.12	0.97	0.84	0.35–2.01	0.70
Beta-blocker	1.05	0.57–1.95	0.88	1.07	0.53–2.13	0.86
Direct renin inhibitor	3.58	0.22–58.07	0.37			
Mineralocorticoid receptor antagonist	0.69	0.23–2.09	0.51			
Class I antiarrhythmic agent	0.31	0.039–2.46	0.27			
Calcium channel blocker	1.56	0.88–2.74	0.13			
Dihydropyridine	1.57	0.88–2.79	0.13			
Non-dihydropyridine	0.81	0.22–2.93	0.75			
Nitric acid	1.28	0.66–2.45	0.47			
Nicorandil	0.97	0.55–1.72	0.92			
Alpha-blocker	3.62	0.50–26.25	0.20			
Diuretic agent	1.00	0.50–2.00	1.00			
Digitalis	1.34	0.35–5.22	0.67			
Anticoagulant agent	0.61	0.24–1.52	0.29			
Warfarin	0.50	0.19–1.33	0.16			
Statin	1.24	0.54–2.83	0.61			
Oral hypoglycaemic agent	0.64	0.35–1.16	0.14			
Insulin	1.47	0.75–2.90	0.26			

Variables that showed a marginal association with LVRR (P < 0.10) in the univariate logistic regression analyses, use of ACE-I/ARB, and use of beta-blocker were adopted as independent variables in the multivariate logistic regression analysis.

*OR* odds ratio; *CI* confidence interval.

## Discussion

From 4394 consecutive patients who underwent PCI, we extracted 286 patients with LV dysfunction (LVEF < 50% at initial LVG), and examined the determinants of LVRR. Multivariate logistic regression analysis revealed that unprotected LMCA intervention, prior PCI, presence of in-stent restenosis at follow-up, and presence of de novo stenosis at follow-up were significant determinants of LVRR after adjusting for confounding factors. Of these factors, unprotected LMCA intervention was the only positive predictor for LVRR. To the best of our knowledge, this is the first study that demonstrated the impact of LMCA intervention on LVRR.

In the present study, the prescription rate of optimal medical therapy including ACE-I/ARB and beta-blockers was similar between patients in the LVRR group and the non-LVRR group. Previous studies have confirmed the efficacy of ACE-I^18,19^, ARB^20^, and beta-blockers^21–23^ on LVRR. There are several confounding factors that may explain these contradictions in optimal medical therapies on LVRR in the present study. The most significant confounding factor was the selection bias for medication, and it is possible that patients with more severe LV dysfunction tended to receive ACE-I/ARB and/or beta-blockers, which may mask the efficacy on LVRR. Furthermore, our study included relatively mild LV dysfunction patients, such as patients with an LVEF near 50% and without symptoms of heart failure, which may weaken the effects of ACE-I/ARB and/or beta-blockers.

Although there was no relationship between aortic systolic pressure and LVRR in multivariate logistic regression analysis in the present study, the univariate logistic regression analysis showed a trend towards a relationship between high aortic systolic pressure and LVRR. Merlo and colleagues previously examined the prognostic role of LVRR and baseline predictors of LVRR in idiopathic dilated cardiomyopathy^4^. In that study, LVRR was significantly associated with prevention of sudden death and major ventricular arrhythmia, and a higher baseline systolic blood pressure was a predictor of LVRR (per 10 mmHg increase; OR 1.23, 95% CI 1.01–1.53, P = 0.047). Furthermore, the authors suggested that patients with higher baseline systolic blood pressure may have more chances of tailoring treatment and modulating the afterload, which may result in LVRR^4^.

Unprotected LMCA disease is associated with high morbidity and mortality because of the large volume of myocardium at risk^24^. Current European and United States guidelines recommend that most patients with unprotected LMCA disease undergo CABG^2,3^. However, randomized trials suggested that PCI with drug-eluting stents (DES), such as sirolimus-eluting stent^12,25^ or paclitaxel-eluting stent^26^, may be an acceptable alternative strategy for selected patients with unprotected LMCA disease. Several meta-analyses have reported similar findings^27–29^. In 2019, the Evaluation of XIENCE versus Coronary Artery Bypass Surgery for Effectiveness of Left Main Revascularization trial revealed that PCI with an everolimus-eluting stent was similar to CABG with respect to the rate of the composite end point of death, stroke, or myocardial infarction at 5 years follow-up in patients with unprotected LMCA disease of low or intermediate anatomical complexity^30^. Nevertheless, the majority of patients in that study had a preserved EF. Accordingly, the association of unprotected LMCA intervention with LVRR remains poorly understood in patients with a reduced EF. The present study focused on the population with LV systolic dysfunction, and found a potential benefit of unprotected LMCA intervention for LVRR. Because LVRR is a significant predictor of long-term outcomes in patients with LV dysfunction^4^, LVRR following PCI may improve long-term clinical outcomes in patients with LV dysfunction if performed together with optimal medical therapy. Currently, there are no randomized trials comparing the effects of PCI and medical therapy with those of medical therapy alone in patients with CAD and LV systolic dysfunction. However, an ongoing trial, REVascularisation for Ischaemic VEntricular Dysfunction (NCT01920048), is comparing the effects of PCI with those of medical therapy alone in patients with CAD and LV systolic dysfunction, and the findings, particularly on the effects of LMCA intervention, are eagerly awaited.

Although the operator’s proficiency such as the left main stent implantation technique may be associated with patient outcomes including LVRR, improvements of DES, anti-platelet therapy, imaging guidance, and physiological lesion assessment have markedly improved PCI outcomes, and the indications of PCI have been expanded to more complex lesions^31^. The present study included left main intervention using a bare-metal stent or 1st generation DES. However, the use of 2nd generation DES may provide a greater improvement in outcomes after unprotected LMCA intervention^32^. Thus, in the future, a larger number of patients with unprotected LMCA disease and LV dysfunction may be treated with PCI, together with improved devices, implantation techniques, and medications^33^.

### Study limitations

As this was a single-centre, retrospective, and observational study, there is a possibility of patient selection bias. LVEF and LV volume were measured using the single plane area-length method by LVG, which is less accurate than biplane ventriculography or cardiac magnetic resonance imaging. Echocardiographic parameters were not included in the analysis because not all patients underwent echocardiography before and after PCI in the echocardiographic laboratory. Patients with an elevated end-diastolic pressure did not receive the LVG examination itself because of an increased risk of acute pulmonary oedema, thereby they were not included into the present study. Although no patients in this study were diagnosed as having severe valvular disease or idiopathic cardiomyopathy, we might not have fully excluded possible pre-existing chronic reduction of LV function, due to other conditions than CAD. It would be better to evaluate myocardial viability using stress scintigraphy before revascularization, however, we did not routinely evaluate myocardial viability by scintigraphy in all cases. Patients with complex CAD were treated with PCI or CABG after the Heart Team discussion, in which anatomical complexity and clinical characteristics were considered, and patients who had LMCA disease and treated with PCI were few in our study population, which might weaken the strength of our findings. Finally, the patients who did not receive PCI were excluded from this study in advance. As the clinical application of PCI basically varies depending upon the lesion type and patient status, this exclusion criteria itself might have caused the patient selection bias.

## Conclusions

Unprotected LMCA intervention was significantly associated with LVRR, while prior PCI, presence of in-stent restenosis, and presence of de novo stenosis were negatively associated with LVRR. These findings suggest a potential benefit of unprotected LMCA intervention for LVRR and importance of angiographic follow-up in patients with CAD and LV systolic dysfunction.

## Supplementary Information

Supplementary Table S1.

## Data Availability

The datasets generated and/or analysed during the current study are available from the corresponding author upon reasonable request.
