# Supplementary material for: Factors associated with left ventricular reverse remodelling after percutaneous coronary intervention in patients with left ventricular systolic dysfunction
Source: Sci Rep. 2021 Jan 8;11:239. doi: 10.1038/s41598-020-80491-y (PMC7794568; doi:10.1038/s41598-020-80491-y)
Supplement: Supplementary file 1 — Supplementary Table S1. [file 41598_2020_80491_MOESM1_ESM.docx]

**Factors Associated with Left Ventricular Reverse Remodelling After Percutaneous Coronary Intervention in Patients with Left Ventricular Systolic Dysfunction**

Yusuke Adachi, MD,^1^ Arihiro Kiyosue, MD,^1*^ Jiro Ando, MD,^1^ Takuya Kawahara, PhD, MPH,^2^ Satoshi Kodera, MD,^1^ Shun Minatsuki, MD,^1^ Hironobu Kikuchi, MD,^1^ Toshiro Inaba, MD,^1^ Hiroyuki Kiriyama, MD,^1^ Kazutoshi Hirose, MD,^1^ Hiroki Shinohara, MD,^1^ Akihito Saito, MD,^1^ Takayuki Fujiwara, MD,^1^ Hironori Hara, MD,^1^ Kazutaka Ueda, MD,^1^ Kenichi Sakakura, MD,^3^ Masaru Hatano, MD,^1,4^ Mutsuo Harada, MD,^1^ Eiki Takimoto, MD,^1^ Hiroshi Akazawa, MD,^1^ Hiroyuki Morita, MD,^1^ Shin-ichi Momomura, MD,^3^ Hideo Fujita, MD,^1,3^ Issei Komuro, MD^1^

^1^Department of Cardiovascular Medicine, Graduate School of Medicine, The University of Tokyo, Tokyo, Japan.

^2^Clinical Research Promotion Center, The University of Tokyo Hospital, Tokyo, Japan.

^3^Division of Cardiovascular Medicine, Saitama Medical Center, Jichi Medical University, Saitama, Japan.

^4^Department of Therapeutic Strategy for Heart Failure, Graduate School of Medicine, The University of Tokyo, Tokyo, Japan.

***** **Address for correspondence:** Dr. Arihiro Kiyosue, Department of Cardiovascular Medicine, Graduate School of Medicine, The University of Tokyo, 7-3-1 Hongo, Bunkyo-ku, Tokyo 113-8655, Japan. E-mail: [kiyosue-tky@umin.ac.jp](mailto:kiyosue-tky@umin.ac.jp)

**Supplemental Table 1.** Medications on admission.

|  | **All**  **(n = 286)** | **LVRR group**  **(n = 63)** | **Non-LVRR group**  **(n = 223)** | **P value** |
| --- | --- | --- | --- | --- |
| ACE-I and/or ARB, n (%) | 198 (69.2) | 41 (65.1) | 157 (70.4) | 0.44 |
| Beta-blocker, n (%) | 142 (49.7) | 28 (44.4) | 114 (51.1) | 0.39 |
| Direct renin inhibitor, n (%) | 2 (0.7) | 1 (1.6) | 1 (0.4) | 0.39 |
| Mineralocorticoid receptor antagonist, n (%) | 20 (7.0) | 2 (3.2) | 18 (8.1) | 0.26 |
| Spironolactone, n (%) | 19 (6.6) | 2 (3.2) | 17 (7.6) | 0.26 |
| Eplerenone, n (%) | 1 (0.3) | 0 (0) | 1 (0.4) | 1.00 |
| Class I antiarrhythmic agent, n (%) | 13 (4.5) | 1 (1.6) | 12 (5.4) | 0.31 |
| Class III antiarrhythmic agent, n (%) | 5 (1.7) | 0 (0) | 5 (2.2) | 0.36 |
| Calcium channel blocker, n (%) | 108 (37.8) | 26 (41.3) | 82 (36.8) | 0.56 |
| Dihydropyridine, n (%) | 93 (32.5) | 25 (39.7) | 68 (30.5) | 0.17 |
| Non-dihydropyridine, n (%) | 17 (5.9) | 1 (1.6) | 16 (7.2) | 0.13 |
| Nitric acid, n (%) | 64 (22.4) | 14 (22.2) | 50 (22.4) | 1.00 |
| Nicorandil, n (%) | 89 (31.1) | 18 (28.6) | 71 (31.8) | 0.65 |
| Alpha-blocker, n (%) | 5 (1.7) | 2 (3.2) | 3 (1.3) | 0.59 |
| Diuretic agent, n (%) | 52 (18.2) | 11 (17.5) | 41 (18.4) | 1.00 |
| Digitalis, n (%) | 10 (3.5) | 3 (4.8) | 7 (3.1) | 0.70 |
| Orally active cardiac stimulant, n (%) | 0 (0) | 0 (0) | 0 (0) | - |
| Anticoagulant agent, n (%) | 29 (10.1) | 3 (4.8) | 26 (11.7) | 0.16 |
| Warfarin, n (%) | 29 (10.1) | 3 (4.8) | 26 (11.7) | 0.16 |
| Direct oral anticoagulant, n (%) | 0 (0) | 0 (0) | 0 (0) | - |
| Statin, n (%) | 193 (67.5) | 37 (58.7) | 156 (70.0) | 0.10 |
| Oral hypoglycaemic agent, n (%) | 101 (35.3) | 16 (25.4) | 85 (38.1) | 0.073 |
| Insulin, n (%) | 52 (18.2) | 13 (20.6) | 39 (17.5) | 0.58 |

Data are expressed as numbers (%). ACE-I, angiotensin converting enzyme inhibitor; ARB, angiotensin II receptor blocker.
